# Engagement of people with lived experience in studies published in high-impact psychiatry journals: meta-research review

**DOI:** 10.1186/s40900-024-00651-6

**Published:** 2024-11-06

**Authors:** Claire Adams, Elsa-Lynn Nassar, Julia Nordlund, Sophie Hu, Danielle B. Rice, Vanessa Cook, Jill Boruff, Brett D. Thombs

**Affiliations:** 1 Lady Davis Institute for Medical Research, Jewish General Hospital, 3775 Cote Ste Catherine Road, Pavilion H4.83, Montreal, QC H3T 1E2 Canada; 2 Department of Psychiatry, McGill University, Montreal, QC Canada; 3 Department of Psychology, McGill University, Montreal, QC Canada; 4 Department of Psychology, St. Joseph’s Healthcare Hamilton, Hamilton, ON Canada; 5 Department of Psychiatry & Behavioural Neurosciences, McMaster University, Hamilton, ON Canada; 6 Schulich Library of Physical Sciences, Life Sciences, and Engineering, McGill University, Montreal, QC Canada; 7 Department of Epidemiology, Biostatistics, and Occupational Health, McGill University, Montreal, QC Canada; 8 Department of Medicine, McGill University, Montreal, QC Canada; 9 Biomedical Ethics Unit, McGill University, Montreal, QC Canada

**Keywords:** Mental health, Methodology, Patient involvement, Public involvement, Proportions, Research partnerships

## Abstract

**Background:**

We evaluated studies published in high-impact psychiatry journals to assess (1) the proportion that reported in articles whether they engaged people with lived experience; (2) the proportion that likely engaged people with lived experience; and, if engagement occurred, (3) stages of research (planning, conduct, interpretation, dissemination); and (4) engagement level (consult, involve, partner).

**Methods:**

We searched PubMed on December 14, 2022, for articles in psychiatry journals with impact factor ≥ 10 and reviewed articles in reverse chronological order until 141 were included, based on pre-study precision estimation. We contacted authors to obtain information on engagement.

**Results:**

Three of 141 (2%) studies reported engagement of people with lived experience in articles. Of the other 138 studies, 74 authors responded to follow-up emails and 22 reported they engaged people with lived experience but did not report in the article. Depending on assumptions about engagement by non-responders, we estimated, overall, 18-31% of studies may have engaged people with lived experience. Engagement occurred in research planning (70%) and rarely interpretation (35%). Most involved consultation (providing opinions or perspectives, 53%) and few involved partnership (11%).

**Conclusions:**

Engagement of people with lived experience in psychiatry research is uncommon, and when it does occur people are typically consulted but not engaged in roles with influence on decision-making. Funding agencies, ethics committees, journals, and academic institutions should take steps to support engagement of people with lived experience in psychiatry research.

**Supplementary Information:**

The online version contains supplementary material available at 10.1186/s40900-024-00651-6.

## Abstract (plain language summary)

Engaging people with lived experience in research is important to ensure research is relevant to people’s needs. We do not know, however, how often people with lived experience are engaged in psychiatry research and whether engagement is reported in journal articles. We evaluated studies published in top psychiatry journals and assessed the (1) percentage that reported in the articles whether they engaged people with lived experience and (2) percentage that likely engaged people with lived experience based on author queries. Additionally, if people with lived experience were engaged in the studies, we also evaluated (3) the stages of research that people with lived experience were involved in (planning, conduct, interpretation, dissemination); and (4) their level of influence on decision-making (consult, involve, partner). Articles published in psychiatry journals with impact factor greater than or equal to 10 were eligible. We searched for studies on PubMed, a popular database of medical research, and reviewed articles from most to least recent until 141 were included. We calculated that we needed to review 141 articles to have enough to make reasonably precise estimates (confidence intervals around estimates of 10% or less) of how many studies engaged people with lived experience. We found that 3 of 141 (2%) studies reported on engagement in articles. We contacted authors of studies that did not report on engagement in their articles and asked whether they engaged people with lived experience. Of the 138 articles that did not report on engagement, 74 authors responded to email queries, and 22 reported they engaged people with lived experience. We estimated, overall, 18-31% of studies may have engaged people with lived experience, depending on whether we assumed that non-responders did or did not engage. Among all studies that engaged people with lived experience, both those reported and unreported in the articles, engagement commonly occurred in research planning (*N* = 14 of 20 studies, 70%) but rarely in the interpretation of results (*N* = 7 of 20 studies, 35%). Engagement mostly involved consultation, where people with lived experience provided perspectives to researchers (*N* = 10 of 19 studies, 53%). Few involved partnership, where they worked with researchers to make decisions (*N* = 2 of 19 studies, 11%). Funding agencies, ethics committees, academic journals, and universities should take steps to support engagement of people with lived experience in psychiatry research.

**Supplementary Information:**

The online version contains supplementary material available at 10.1186/s40900-024-00651-6.

## Background

**Fig. 1:**
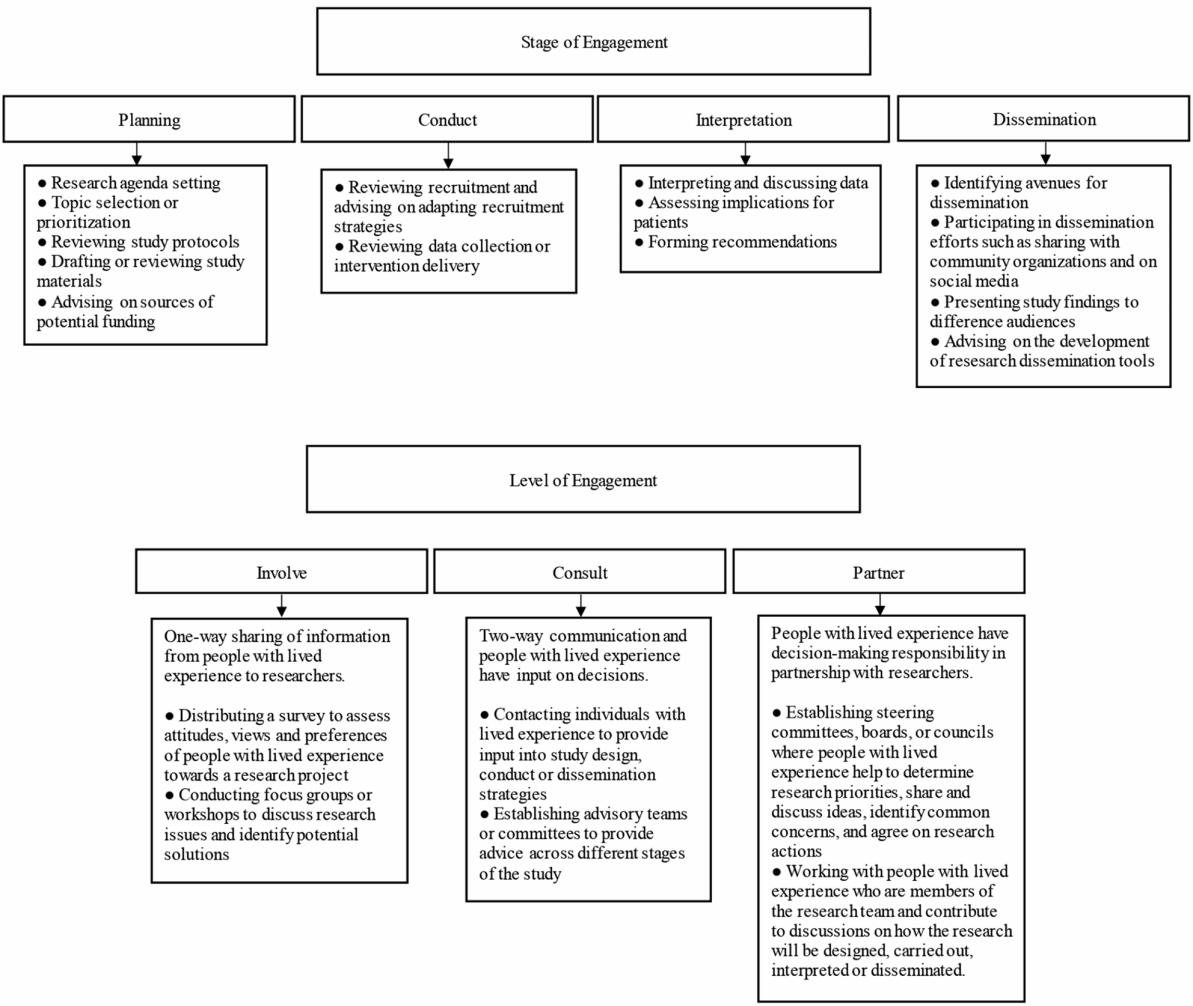
Engagement of People with Lived Experience in Research by Stage and Level of Engagement

Engagement of people with lived experience in health research involves active, meaningful collaboration between people with lived experience and researchers in research planning, conduct, interpretation, or dissemination [1]. Engagement may occur via [1] consultation and contribution of perspectives on planned or ongoing research; [2] involvement in a research team and advising or making recommendations; 3) partnering with researchers, including in decision-making [2–4]. Figure 1 shows examples of ways people with lived experience engage across research stages and engagement levels.

Engaging people with lived experience improves research relevance, quality, and translation, and increases accountability [5, 6]. Engagement in mental research in particular can help to prioritise research questions and outcomes, improve recruitment processes, improve adherence to study procedures, and enhance dissemination [7, 8]. Historically, people with mental health challenges were disempowered by government systems and research, and barriers such as mental health stigma and symptoms of mental illness (e.g., cognitive, psychological) create additional challenges to engagement that can result in tokenistic engagement or omission of patient views completely [9, 10]. Engagement in research is a way people with lived experience can regain their voice and advocate for their needs. Ensuring meaningful engagement of people with lived experience in mental health research is especially important to address inequalities and address unmet needs [10]. Many national-level funding agencies encourage or mandate patient and public engagement and have developed engagement principles, frameworks, or guidance to increase engagement of people with lived experience in health research [11–16]. 

In January 2024, Lancet Psychiatry became the first high-impact psychiatry journal (impact factor ≥10) to require submitted manuscripts to include a statement about involvement of people with lived experience [17]. No studies, however, have examined engagement and reporting of engagement of people with lived experience in psychiatry journals. Two previous studies explored the engagement of people with lived experience in mental health research. But, these studies only examined only one journal or one country-specific database and thus do not tell us the proportion of studies that engaged people with lived experience and reporting of engagement across the field of psychiatry [7, 18]. Another study scoped the literature on service user involvement in mental health research, but only studies that engaged mental health service users or their relatives were included to determine how engagement occurred, not how common it is [19]. There is a need to better understand how often people with lived experience are engaged across psychiatry research and whether engagement is reported in journal articles to ensure research in the field is relevant to people’s needs.

We reviewed primary human research studies in high-impact psychiatry journals and evaluated: (1) the proportion that reported in articles whether they engaged people with lived experience and (2) the proportion that likely engaged people with lived experience, including engagement reported in articles and not reported in articles, based on author queries. If people with lived experience were engaged in the studies we also evaluated, (3) the stages of research that people with lived experience were engaged in (planning, conduct, interpretation, dissemination), and (4) their level of influence on decision-making (consult, involve, partner).

## Methods

We reviewed recently published primary human research studies in high-impact psychiatry journals to evaluate engagement practices of people with lived experience and reporting of engagement. We posted a study protocol (https://osf.io/bh2qw) prior to study initiation.

### Identification of eligible studies

Journals included in the Clarivate Journal Citation Reports™ category *Psychiatry* with 2021 impact factor ≥ 10 were eligible. We chose the category *Psychiatry*, as this is the category most directly related to mental health. Other categories, such as psychology, include research over broad areas (e.g., development, education, biology) less consistently relevant to our objectives. We required impact factor ≥ 10 to select studies in influential journals likely to have resources to engage people with lived experiences. See supplement (p 2) for eligible journals.

We defined people with lived experience as those directly affected by mental ill health, which could include patients, carers, family members, friends or communities. We defined primary human research studies as studies that used data collected by study authors for research. We excluded studies that used clinical records without requiring patient consent, data from clinical or quality control registries, health care administration databases, national census databases, shared databases collected by other researchers, and biobanks, as these studies do not require primary data collection and may be more limited in how people with lived experiences are engaged. We excluded systematic reviews and meta-analyses. We excluded research letters and brief or concise reports due to restrictions on word counts that limit the ability to describe engagement.

All included journals are fully cited in PubMed. Thus, we searched PubMed for eligible studies on December 14, 2022, from inception to present, using a systematic search designed by an experienced health sciences librarian. See supplement (p 3) for the search strategy.

### Selection of eligible studies

To include the most recently published studies, we reviewed citations in reverse chronological order based on PubMed Unique Identifiers until we obtained our targeted sample size. We included only recently published studies as the engagement of people with lived experience in research is evolving and we wanted to evaluate current practices. The PubMed search was conducted via DistillerSR systematic review software (Evidence Partners, Ottawa, Canada), and citations were uploaded into the platform. Two investigators independently assessed study eligibility at the title and abstract level. If either investigator considered a study potentially eligible, it proceeded to full-text review, where it was further assessed for eligibility, independently by two investigators. Conflicts were resolved by consensus, with a third reviewer consulted as necessary. See supplement (pp 4–5) for screening forms.

### Sample size

We calculated sample size to ensure that our estimates of proportions would be sufficiently precise to be useful. Our experience, prior to initiating this study, suggested that few studies in psychiatry journals report on engagement of people with lived experience. We estimated the proportion would likely be < 10%. Thus, we set our sample size to have a 95% confidence interval (CI) width of 10% around a percentage reporting of 10%. Based on CIs calculated using Agresti and Coull’s method [20], we aimed to obtain 141 studies, and we determined that even if up to 30% reported, 141 studies would still generate a 95% CI width of 15%.

### Data extraction

At the time searches were conducted, for each eligible journal, two reviewers independently reviewed the journal’s website, including author instructions, and coded whether journals encouraged or required reporting of engagement of people with lived experience in manuscripts.

For each included primary study, we extracted: (1) article information, including first author’s surname; publication month and year, with “online ahead of print” designations subsequently updated to final publication month and year; journal name; funding source(s); country of corresponding author; country or countries where study participants were recruited; (2) study design; (3) study aim; (4) recruitment setting; study population; number of participants; (5) study topic (e.g., interventions; outcomes or prognosis); (6) whether engagement of people with lived experience was reported in the article; if yes (7), all of the stages of the research process in which people with lived experience were engaged (research planning; conduct; interpretation; dissemination); (8) the level in which people with lived experience were engaged (consult; involve; partner). We defined stages and engagement levels based on prominent international and national frameworks on engagement [11–16]. Data were extracted by one investigator in DistillerSR and validated by a second investigator using the DistillerSR Quality Control function. Conflicts were resolved by consensus, with a third investigator consulted as necessary. See supplement (pp 6–10) for the data extraction form, including coding guides for stages and levels.

For studies that did not report whether people with lived experience were engaged or the stage or level of engagement, we emailed corresponding authors to attempt to obtain this information. Authors were invited to complete a form in the online survey platform Qualtrics to describe if patients or the public were engaged in their study and, if so, stage and engagement level based on descriptions we provided. See supplement (pp 11–13) for email template and form. We emailed corresponding authors once per week, up to three weeks, until a response was received. At the third week, if there was no response, we emailed study co-authors. In instances where the email for the corresponding author did not function, we searched for an alternate email address or emailed co-authors.

### Involvement of people with lived experience

Because of the bibliographic or meta-research focus of this study, people with lived experience of mental ill health were not involved in its design, conduct, interpretation, or dissemination. Members of the authorship team have experience as researchers, editors, and in funding policy and decision-making.

### Analysis

We recorded the proportion of included studies that reported in articles whether people with lived experience were engaged. Among studies that did not report on engagement in the articles, we recorded the proportion of authors that completed our survey and had engaged people with lived experience, completed our survey and had not engaged people with lived experience, and did not respond. We estimated the overall proportion of engagement of people with lived experience based on two assumptions: (1) that authors who did not respond did not engage people with lived experience; (2) that the same proportion of authors who did and did not respond engaged people with lived experience.

Among studies that engaged people with lived experience, we determined the proportion that engaged at each stage of the research process (planning, conduct, interpretation, dissemination) and the proportion that engaged at each level (consult, involve, partner). All proportions are presented with 95% CIs using the method of Agresti and Coull [20]. 

Further to main results, we presented results by subgroups defined by region or country, study design, population, and topic. Subgroups were established a posteriori based on frequency data. We did not conduct statistical tests to compare subgroups because our study was not designed or powered for that purpose.

## Results

**Fig. 2:**
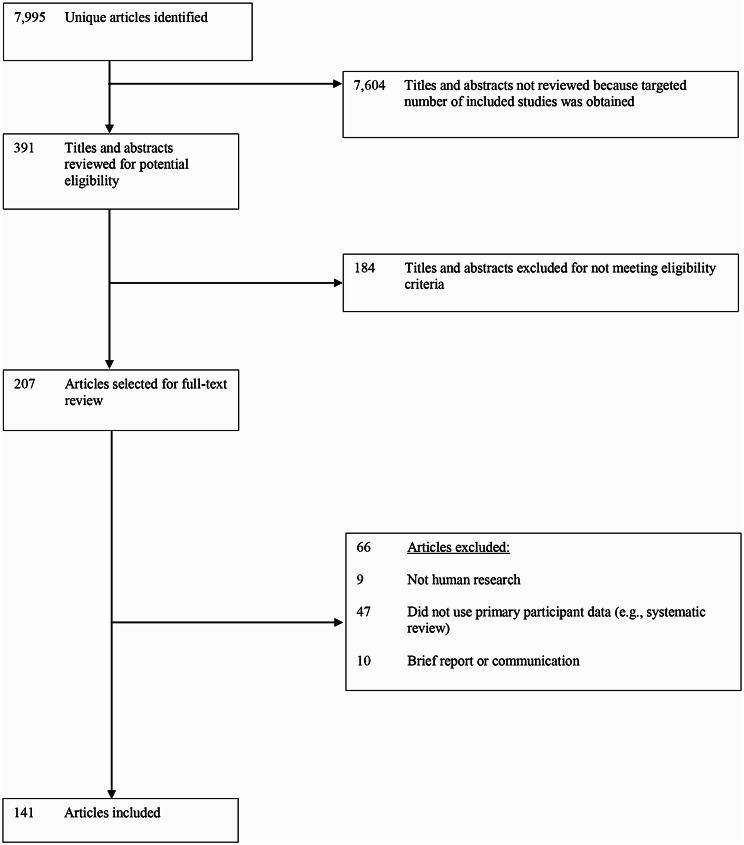
PRISMA flow diagram

Our search yielded 7,995 unique titles and abstracts. We excluded 184 titles and abstracts and 66 full-texts, reviewing in reverse chronological order, until we obtained 141 included studies. See Fig. 2. Included studies were first published online between October 2022 to December 2022, with final publication dates from October 2022 to October 2023. Journals with ten or more included studies were Psychiatry Research (*N* = 27), Psychological Medicine (*N* = 24), Molecular Psychiatry (*N* = 15), the Asian Journal of Psychiatry (*N* = 13), the International Journal of Social Psychiatry (*N* = 13), and the Journal of Neurology Neurosurgery and Psychiatry (*N* = 11). See supplement (p 2). No eligible journals encouraged or required reporting of engagement of people with lived experience in author instructions.

As shown in Table 1, most corresponding author affiliations were from Europe (*N* = 54, 38%), North America (*N* = 38, 27%), and China (*N* = 24, 17%). Most studies reported non-industry funding (*N* = 117, 83%). Most studies were cross-sectional observational (*N* = 58, 41%), longitudinal cohort (*N* = 36, 26%), or interventional studies (*N* = 34, 24%). Most included non-psychiatric population samples (*N* = 43, 31%) or participants with severe (*N* = 30, 21%) or common (*N* = 27, 19%) mental disorders. Research topics included factors associated with mental disorders, prognosis of mental disorders, or predictors of intervention effects (*N* = 48, 34%), neuroimaging, genetics, or biomarkers studies (*N* = 42, 30%), and intervention effects (*N* = 30, 21%). See supplement (p 14) for characteristics and results of all included studies.

**Table 1:** Study characteristics (*N* = 141)

Study Characteristics	*N* (%)	
**Region or Country of Corresponding Author**		
Europe ^a^	54 (38%)	
North America^b^	38 (27%)	
China	24 (17%)	
Other Asian countries^c^	16 (11%)	
Other countries^d^	9 (7%)	
**Funding Source**		
Non-industry	117 (83%)	
International or national funding agency only	52 (45%)	
State or provincial funding agency only	4 (3%)	
University or hospital funding agency only	5 (4%)	
Other not-for-profits only	8 (7%)	
Multiple non-industry funders	48 (41%)	
Industry	2 (1%)	
No funding	19 (14%)	
Not reported	3 (2%)	
**Study Design**		
Cross-sectional observational study	58 (41%)	
Longitudinal cohort study	36 (26%)	
Intervention trial^e^	34 (24%)	
Case-control study	6 (4%)	
Diagnostic assessment study	5 (4%)	
Other^f^	2 (1%)	
**Recruitment Setting**		
Outpatient or community clinics	31 (22%)	
Internet	14 (10%)	
Hospital inpatient	12 (9%)	
Pre-existing cohorts	9 (7%)	
University campus	7 (5%)	
Hospital (clinic unspecified)	5 (3%)	
Multiple settings	30 (21%)	
Other^g^	17 (12%)	
Unclear or not reported	16 (11%)	
**Population**		
Non-psychiatric	43 (31%)	
Severe mental disorders (e.g., psychosis, schizophrenia)	30 (21%)	
Common mental disorders (e.g., anxiety, depression)	27 (19%)	
Neurological disorders (e.g., Alzheimer’s, epilepsy)	11 (8%)	
At risk of mental disorders (e.g., refugees, people who experienced a recent loss or traumatic event)	10 (7%)	
Neurodevelopmental disorders (e.g., autism, attention deficit)	9 (6%)	
Other disorders^h^	11 (8%)	
**Study Topic**		
Factors associated with mental disorders, prognosis of mental disorders, predictors of intervention outcomes	48 (34%)	
Neuroimaging, genetics, or biomarkers	42 (30%)	
Intervention effects^i^	30 (21%)	
Other^j^	21 (15%)	

^a^Belgium (1); Croatia (1); Denmark (4); Finland (1); France (3); Germany (8); Italy (8); the Netherlands (6); Norway (3); Poland (1); Spain (6); Sweden (1); United Kingdom (11); ^b^Canada (8); United States of America (29); United States of America and Canada (1); ^c^India (7); Israel (2); Malaysia (2); South Korea (2); Taiwan (3); ^d^Australia (5); Brazil (1); Egypt (1); New Zealand (1); United Kingdom and China (1);^e^Includes randomised and non-randomised controlled trials. ^f^Qualitative study (1); Experimental design where some cells, but not others, were exposed to alcohol (1). ^g^Basic combat training, United States Army (1); Community (4); Mailing lists, databases or registers (5); Old age homes (1); Refugee camps (2); Schools (3); Semi-hospitalization program (1); ^h^Eating disorders (2); Multiple mental disorders (5); Neurodegenerative disorders (1); Substance use disorders (3); ^i^Topics were coded based on what was deemed primary by the authors of the manuscript. Four studies involved secondary analyses from trials and reported factors associated with outcomes, and thus were coded as factors studies. ^j^Attitudinal study (2); COVID-19 study (1); Lived experiences study (1); Mental health measurement or screening tool development or validation study (5); Mental health services research (1); Prevalence or burden of mental disorders study (7); Trajectory of mental health symptoms or diagnoses over time (4)

### Proportion of studies that engaged people with lived experience

As shown in Table 2, three of 141 included studies (2%; 95% CI 1–6%) mentioned engagement of people with lived experience in the journal article [21–23]. None of the three articles indicated that they included people with lived experience as co-authors, and all co-authors had academic affilitations.

**Table 2:** Number and percent of studies that engaged people with lived experience, reported in the article or based on author email responses, for the overall sample and subgroups

	Engagement Reported in the Article (*N* = 141)		Engagement not reported in the Article (*N* = 138)			Estimated Engagement Among all Articles (*N* = 141)	
	Engaged People with Lived Experience *N* (%; 95% CI)	Did not Report *N* (%; 95% CI)	Engaged People with Lived Experience: per Author Report *N* (%)	Did not Engage People with Lived Experience: per Author Report *N* (%)	Authors Did not Respond *N* (%)	Assume Non-responders did not Engage *N* (%; 95% CI)	Assume Same Proportion of Non-responders Engaged as Responders *N* (%; 95% CI)
**All Studies**	3 (2%; 1%, 6%)	138 (98%; 94%, 99%)	22 (16%)	52 (38%)	64 (46%)	25 (18%; 12%, 25%)	44 (31%; 24%, 39%)
**Region or Country**							
Europe	2 (4%; 1%, 13%)	52 (96%; 87%, 99%)	12 (23%)	19 (37%)	21 (40%)	14 (26%; 16%, 39%)	22 (41%; 29%, 54%)
North America	0 (0%; 0%, 9%)	38 (100%; 91%, 100%)	2 (5%)	16 (42%)	20 (53%)	2 (5%; 1%, 17%)	4 (11%; 4%, 24%)
China	1 (4%; 1%, 20%)	23 (96%; 80%, 99%)	4 (17%)	5 (22%)	14 (61%)	5 (21%; 9%, 40%)	11 (46%; 28%, 65%)
Other Asian countries	0 (0%; 0%, 19%)	16 (100%; 81%, 100%)	3 (19%)	8 (50%)	5 (31%)	3 (19%; 7%, 43%)	4 (25%; 10%, 49%)
Other countries	0 (0%; 0%, 30%)	9 (100%; 70%, 100%)	1 (11%)	4 (44%)	4 (44%)	1 (11%; 2%, 44%)	2 (22%; 6%, 55%)
**Study Design**							
Cross-sectional observational study	2 (3%; 1%, 12%)	56 (97%; 88%, 99%)	10 (18%)	19 (34%)	27 (48%)	12 (21%; 12%, 33%)	21 (36%; 25%, 49%)
Longitudinal cohort study	1 (3%; 0%, 14%)	35 (97%; 86%, 100%)	4 (11%)	15 (43%)	16 (46%)	5 (14%; 6%, 29%)	8 (22%; 12%, 38%)
Intervention trial	0 (0%; 0%, 10%)	34 (100%; 90%, 100%)	5 (15%)	14 (41%)	15 (44%)	5 (15%; 6%, 30%)	9 (26%; 15%, 43%)
Case-control study	0 (0%; 0%, 39%)	6 (100%; 61%, 100%)	2 (33%)	0 (0%)	4 (67%)	2 (33%; 10%, 70%)	6 (100%; 61%, 100%)
Diagnostic assessment study	0 (0%; 0%, 43%)	5 (100%; 57%, 100%)	1 (20%)	2 (40%)	2 (40%)	1 (20%; 4%, 62%)	2 (40%; 12%, 77%)
Other study design	0 (0%; 0%, 66%)	2 (100%; 34%, 100%)	0 (0%)	2 (100%)	0 (0%)	0 (0%; 0%, 66%)	0 (0%; 0%, 66%)
**Population**							
Non-psychiatric population	1 (2%; 0%, 12%)	42 (98%; 88%, 100%)	8 (19%)	17 (40%)	17 (40%)	9 (21%; 11%, 35%)	14 (33%; 20%, 47%)
People with severe mental disorders	1 (3%; 1%, 17%)	29 (97%; 83%, 99%)	7 (24%)	10 (34%)	12 (41%)	8 (27%; 14%, 44%)	13 (43%; 27%, 61%)
People with common mental disorders	0 (0%; 0%, 12%)	27 (100%; 88%, 100%)	3 (11%)	10 (37%)	14 (52%)	3 (11%; 4%, 28%)	6 (22%; 11%, 41%)
People with neurological disorders	0 (0%; 0%, 26%)	11 (100%; 74%, 100%)	0 (0%)	4 (36%)	7 (64%)	0 (0%; 0%, 26%)	0 (0%; 0%, 26%)
People at risk of mental disorders	0 (0%; 0%, 28%)	10 (100%; 72%, 100%)	2 (20%)	5 (50%)	3 (30%)	2 (20%; 6%, 51%)	3 (30%; 11%, 60%)
People with neurodevelopmental disorders	0 (0%; 0%, 30%)	9 (100%; 70%, 100%)	1 (11%)	1 (11%)	7 (78%)	1 (11%; 2%, 44%)	5 (56%; 27%, 81%)
Other disorders	1 (9%; 2%, 38%)	10 (91%; 62%, 98%)	1 (10%)	5 (50%)	4 (40%)	2 (18%; 5%, 48%)	3 (27%; 10%, 57%)
**Study Topic**							
Other factors associated with mental disorders or with prognosis of mental disorders	3 (6%; 2%, 17%)	45 (94%; 83%, 98%)	8 (18%)	16 (36%)	21 (47%)	11 (23%; 13%, 37%)	18 (38%; 25%, 52%)
Neuroimaging, genetics, or biomarkers study	0 (0%; 0%, 8%)	42 (100%; 92%, 100%)	4 (10%)	15 (36%)	23 (55%)	4 (10%; 4%, 22%)	9 (21%; 12%, 36%)
Effects of mental health interventions	0 (0%; 0%, 11%)	30 (100%; 89%, 100%)	4 (13%)	12 (40%)	14 (47%)	4 (13%; 5%, 30%)	8 (27%; 14%, 44%)
Other topic	0 (0%; 0%, 15%)	21 (100%; 85%, 100%)	6 (29%)	9 (43%)	6 (29%)	6 (29%; 14%, 50%)	8 (38%; 21%, 59%)

CI = confidence interval

Among authors of the 138 studies that did not report whether they engaged people with lived experience, 74 responded to our email (54%); of these, 22 (30%) reported that they engaged people with lived experience, and 52 (70%) reported that they did not. We estimated that, overall, 18% (95% CI 12–25%) to 31% (95% CI 24–39%) of the 141 included studies engaged people with lived experience, depending on whether non-responders were assumed to have not engaged people with lived experience or if we assumed they engaged in the same proportion as responders.

Although sample sizes per subgroup were small, studies conducted by authors in Europe (estimated 26–41%) or China (estimated 21–46%) appear to have been more likely to engage than studies conducted in North America (estimated 5–11%). By population, the highest estimated proportion of studies that likely engaged occurred among studies on people with severe mental disorders (27–43%). Among study designs, longitudinal cohort studies (estimated 14–22%) and intervention trials (15–26%) may have lower engagement than other study types. See supplement (p 15) for engagement by journal.

### Stages and levels of engagement of people with lived experience

Among the 25 of 141 included studies (18%) that reported that they engaged people with lived experience, either in the article or in response to our email, 20 provided data on engagement stage, and 19 provided data on engagement level. Of the 20 that reported on engagement stage, 14 (70%; 95% CI 48–85%) engaged people with lived experience in research planning, 13 in research conduct (65%; 95% CI 43–82%), seven in results interpretation (35%; 95% CI 18–57%), and 11 in research dissemination (55%; 95% CI 34–74%). Of the 19 that reported on engagement level, ten (53%; 95% CI 32–73%) engaged at the consult level, where people with lived experience provided opinions or perspectives on research activities; seven (37%; 95% CI 19–59%) engaged at the involve level, where people with lived experience collaborated with researchers to advise and provide recommendations; and two (11%; 95% CI 3–31%) engaged at the partner level, where people with lived experience worked in partnership with researchers to undertake research and make decisions. Full results, including for subgroups, are shown in the supplement (p 16).

## Discussion

We examined 141 articles on primary human research published in high-impact psychiatry journals. Of these, three (2%) reported in their articles that they engaged people with lived experience. Of the other 138, 74 authors responded to our email; 22 (30%) indicated they engaged people with lived experience, and 52 (70%) indicated they did not. Overall, we estimated the proportion of studies that engaged people with lived experience may be between 18% and 31%. Studies conducted in Europe and China may be more likely to engage people with lived experience than studies conducted in North America. Research to improve engagement practices was included in a 2014 European Commission funded roadmap for mental health research [24, 25], and it is possible that this may have contributed to what appear to be somewhat higher rates of engagement in Europe. We evaluated different study types, including imaging, genetics, or biomarkers studies. Engagement in intervention trials, which may be the most directly relevant type of study for people with lived experience, was among the lowest by study design, suggesting the mix of study types in our pool of included studies was not likely the reason for the overall low engagement we found.

Of the 25 studies that engaged people with lived experience, 20 provided data on engagement stage, and 19 provided data on engagement level. People with lived experience were most commonly engaged in research planning (70%), followed by research conduct (65%), research dissemination (55%), and results interpretation (35%). Most studies engaged people with lived experience at the consult level (53%), followed by involve (37%), and partner (11%).

Our study is the first, to our knowledge, to estimate the proportion of reported and actual engagement of people with lived experience in mental health research studies published in top journals. We found that engagement was not standard practice. We estimated that, at best, about 1/3 of included studies may engage people with lived experience, most commonly at the lowest level of engagement, consult. This is despite engagement of people with lived experience being promoted for over two decades by major funders, including, for example, the Canadian Institutes of Health Research, which developed a framework for citizen engagement in 2008; [2] the United Kingdom National Institute for Health Research, which established INVOLVE in 1996 to support public involvement in research; [14] and the establishment of agencies to promote consumer-oriented research and involvement, such as Australia’s Consumer and Community Involvement Program, established in 1998; [26] and the United States Patient-Centered Outcomes Research Institute, established in 2010 [15]. 

We were able to identify two previous studies that examined rates of engagement of people with lived experience in mental health research. A 2022 study examined 3000 articles published in 2020 in a general health journal, *BMJ Open*, which requires a statement on patient and public involvement; it included 149 articles on mental health and found that 55 (37%) were likely to have engaged patients or the public. It did not, however, examine the stages of research where engagement occurred or level of engagement [18]. A 2013 longitudinal study examined 374 articles published in the Mental Health Research Network database, a large database of mental health studies published since 2004, funded by the United Kingdom National Institute for Health Research or its partners [7]. Patient engagement was found to increase over time, however, increases were limited, particularly among groups with complex needs, such as personality and developmental disorders, and the proportion of studies that engaged patients was not reported. As in our review, consult was the most common level of engagement across all studies regardless of funding type. The research network itself, however, emphasises and supports patient engagement in research, so these results may not be representative of mental health research, broadly.

We recently examined engagement of people with lived experience in rheumatology research using methods similar to those of the present study. We found that 9% of the 141 studies we included engaged people with lived experience, which is higher than the 2% we found in psychiatry research [27]. We estimated the proportion of studies that engaged people with lived experience in rheumatology research may be between 29% and 44% based on author queries and the same assumptions as in the present study where we estimated 18–31%. There are barriers to engaging people with lived experience in mental health research. Symptoms of mental disorders, such as cognitive limitations, anxiety, fatigue, and mental health stigma, create challenges to engagement beyond what may occur in other research fields [9]. Practical issues such as time, budget, and recruitment also make engagement challenging in some settings [28]. A 2019 scoping review identified 32 studies that explored how patients were engaged in mental health research and challenges to engagement [19]. Most studies were published between 2000 and 2017 (97%), the majority from the United Kingdom (56%). Among included studies, 53% were designed specifically to facilitate collaboration between researchers and patients; however, important challenges were documented including unclear expectations regarding roles and tasks and a lack of knowledge on how to build equal partnerships. The results of our present study, though they show that engagement is limited, are encouraging. Studies that involved people with severe mental disorders were the most likely to engage patients, which provides evidence that barriers can be overcome.

Increasing engagement of people with lived experience in mental health research will require concrete action by funding agencies, ethics boards, journal publishers, and universities to support researchers to engage people with lived experience and to report on engagement. National and international funding agencies can mandate engagement in their funding applications, allocate funds for researchers to implement engagement in their research, and monitor implementation. To support this, funders should provide education plus practical tools and resources to help researchers plan and implement engagement of people with lived experience. For example, the Canadian Institutes of Health Research’s (CIHR) Institute of Musculoskeletal Health and Arthritis (IMHA) provides free, online, self-guided patient engagement training for researchers looking to engage patients in their research [29]. The Patient-Centered Outcomes Research Institute (PCORI) in the United States has developed a free self-paced online training package to help people new to patient-centered health research to understand research processes and engagement as well as a resource repository of engagement-related tools and guides [30]. Resources tailored to mental health research would help bridge this gap.

Ethics committees can adopt policies that emphasise engagement of people with lived experience as an ethical obligation, and local ethics boards can require or strongly encourage engagement as part of consideration of ethical approval of proposed projects. Canadian national research ethics policy, for example, encourages researchers to engage with the community across study designs and populations and suggests ethics boards may require engagement [31]. We do not, however, know of examples in which ethics committees have implemented and enforced engagement polices.

Journals could consider requiring engagement statements in manuscript submissions to improve reporting. In January 2024, the *Lancet Psychiatry* announced that, going forward, they will be requesting that authors report on involvement of people with lived experience in their manuscripts [17]. *Lancet Psychiatry* is the first of all journals included in our review to encourage or require reporting on engagement, and our study provides a useful benchmark that will be helpful for evaluating how involvement of people with lived experience evolves in studies in the journal and other top psychiatry journals.

University faculties can also promote engagement by reviewing studies that engage people with lived experience positively in tenure and promotion decisions. A recent study of 92 leading international universities found traditional criteria for promotion and tenure used by most (e.g., publication numbers, authorship order, journal impact factors, grant funding) may discourage research that best supports the public good. The authors argued that non-traditional criteria such as data sharing, study pre-registration, and adherence to reporting guidelines, all of which support transparent, verifiable, and replicable research should be considered and incentivised [32]. Engagement of people with lived experience similarly fits these goals; effective and meaningful engagement can improve research quality and translation and fulfils obligations to the public, which supports academic research [5, 6]. 

### Strengths and limitations

Our study has important strengths. It is the first to address the degree that mental health studies published in top psychiatry journals engage people with lived experience and report on engagement. We developed and tested study procedures before commencing and published a protocol to support transparency. We contacted authors of included studies to ensure we accurately reported and coded engagement. Additionally, we included studies initially published online between October and December 2022, which allowed us to draw conclusions about recent practices. There are also limitations. First, we only included high-impact journals, which could limit generalisability. However, we do not believe that engagement of people with lived experience would likely be higher in lower impact journals where authors often have less funding and resources to engage patients [33]. Thus, our findings may represent a best-case scenario for the current state of engagement in psychiatry research. However, this may not be the case, and further research should include a broader sample of journals. Second, we focused on journals in the Clarivate Journal Citation Reports™ category *Psychiatry* and did not assess the degree that individual studies focused on topics core or peripheral to psychiatry (e.g., studies in the Journal of Neurology, Neurosurgery, and Psychiatry). Third, we did not sample articles per journal; instead, we sampled by recency across high-impact journals, which resulted in more articles from journals that publish issues more frequently or more recently. Proportions of studies with patient engagement may differ between journals, depending on their aims and scope. For example, seven of 13 articles from the *International Journal of Social Psychiatry*, which focuses on social factors that influence mental health and wellbeing, reported that people with lived experience had been engaged, which was notably more than other journals. Fourth, we did not have enough included studies to conduct hypothesis tests by subgroups, but we did not find any indication that there were subgroups with particularly high proportions of engagement. Fifth, people with lived experience of mental ill health were not involved in the present research. It is possible that their involvement could have informed how we designed the study or our interpretation of results.

## Conclusions

Engaging people with lived experience in research improves research quality and relevance and encourages public support of research as critical for solving important health problems. Yet, engagement in mental health research published in high-impact psychiatry journals is low. Only 2% of included studies reported engaging people with lived experience, and we estimated that only approximately 18–31% likely engage people with lived experience. Improving engagement in mental health research will likely require steps from research funders, ethics committees, research institutions, and journals to incentivise or require reporting of engagement.

## Electronic Supplementary Material

Below is the link to the electronic supplementary material.

Supplementary Material 1

## Data Availability

All data generated or analyzed during this study are included in this published article and its supplementary information files.

## Abbreviations

CI: Confidence Interval
