# Supplementary material for: Engagement of people with lived experience in studies published in high-impact psychiatry journals: meta-research review
Source: Res Involv Engagem. 2024 Nov 6;10:115. doi: 10.1186/s40900-024-00651-6 (PMC11539569; doi:10.1186/s40900-024-00651-6)
Supplement: Supplementary file 1 — Supplementary Material 1 [file 40900_2024_651_MOESM1_ESM.docx]

**SUPPLEMENTARY MATERIAL**

**Supplement 1: Eligible Journals in Clarivate Journal Citation Reports^TM^ Category *Psychiatry* with 2021 Impact Factor ≥10**

**Supplement 2: PubMed Search Strategy**

**Supplement 3: Inclusion and Exclusion Criteria Screening Forms**

**Supplement 4: Data Extraction Form**

**Supplement 5: Emails to Authors Templates**

**Supplement 6: Characteristics of Individual Studies**

**Supplement 7: Number of Studies that Engaged People with Lived Experiences, Reported in the Article or Based on Author Email Responses, for the Eligible Journals**

**Supplement 8: Number and Percent of Studies that Engaged People with Lived Experience, Reported and Unreported in the Article, by Stages of Engagement and Levels of Engagement**

**Supplement 1: Eligible Journals in Clarivate Journal Citation Reports^TM^ Category *Psychiatry* with 2021 Impact Factor ≥10**

| **Journal Title** | **2021 Impact Factor** | **Studies Included**  **N (%)** |
| --- | --- | --- |
| World Psychiatry | 79·7 | 0 (0%) |
| Lancet Psychiatry | 77·1 | 3 (2%) |
| JAMA Psychiatry | 25·9 | 4 (3%) |
| Psychotherapy and Psychosomatics | 25·6 | 1 (1%) |
| American Journal of Psychiatry | 19·2 | 2 (1%) |
| Brain Behavior and Immunity | 19·2 | 3 (2%) |
| Asian Journal of Psychiatry | 13·9 | 13 (9%) |
| Journal of Anxiety Disorders | 13·7 | 4 (3%) |
| Journal of Neurology Neurosurgery and Psychiatry | 13·6 | 11 (8%) |
| Evidence-based Mental Health^a^ | 13·5 | 0 (0%) |
| Molecular Psychiatry | 13·4 | 15 (11%) |
| Journal of the American Academy of Child and Adolescent Psychiatry | 13·1 | 1 (1%) |
| Biological Psychiatry | 12·8 | 6 (4%) |
| Neuropsychobiology | 12·3 | 5 (4%) |
| Psychiatry and Clinical Neurosciences | 12·1 | 7 (5%) |
| International Journal of Mental Health and Addiction ^b^ | 11·6 | - |
| Psychiatry Research | 11·2 | 27 (19%) |
| British Journal of Psychiatry | 10·7 | 2 (1%) |
| Psychological Medicine | 10·6 | 24 (17%) |
| International Journal of Social Psychiatry | 10·5 | 13 (9%) |

^a^Now called BMJ Mental Health. ^b^Not included as not all citations are in PubMed.

**Supplement 2: PubMed Search Strategy**

(Adaptive Clinical Trial[Publication Type] OR Clinical Study[Publication Type] OR Clinical Trial[Publication Type] OR Clinical Trial Protocol[Publication Type] OR Clinical Trial, Phase I[Publication Type] OR Clinical Trial, Phase II[Publication Type] OR Clinical Trial, Phase III[Publication Type] OR Clinical Trial, Phase IV[Publication Type] OR Comparative Study[Publication Type] OR Controlled Clinical Trial[Publication Type] OR Equivalence Trial[Publication Type] OR Evaluation Study[Publication Type] OR Journal Article[Publication Type] OR Multicenter Study[Publication Type] OR Observational Study[Publication Type] OR Pragmatic Clinical Trial[Publication Type] OR Randomized Controlled Trial[Publication Type] OR Twin Study[Publication Type] OR Validation Study[Publication Type]) AND ((("The International Journal of Social Psychiatry"[Journal]) OR ("The American Journal of Psychiatry"[Journal])) OR ("The British Journal of Psychiatry: the Journal of Mental Science"[Journal]) OR “International Journal of Mental Health and Addiction [journal] OR "World Psychiatry" [journal] OR "Lancet Psychiatry" [journal] OR "JAMA Psychiatry" [journal] OR "Psychotherapy and Psychosomatics" [journal] OR "Brain, Behavior, and Immunity" [journal] OR "Asian Journal of Psychiatry" [journal] OR "Journal of Anxiety Disorders" [journal] OR "Journal of Neurology Neurosurgery and Psychiatry" [journal] OR "Evidence-based Mental Health" [journal] OR "Molecular Psychiatry" [journal] OR "Journal of the American Academy of Child and Adolescent Psychiatry" [journal] OR "Biological Psychiatry" [journal] OR "Neuropsychobiology" [journal] OR "Psychiatry and Clinical Neurosciences" [journal] OR "Psychiatry Research" [journal] OR "Psychological Medicine" [journal])

**Supplement 3: Inclusion and Exclusion Criteria Screening Forms**

**Title and abstract inclusion and exclusion criteria screening form**

**No: not human research.** Exclude if it is clear from the title or abstract that the article does not report on human research.

**No: does not use primary data.** Exclude if the title or abstract of the article indicates the article does not report on a study that used primary data collection, defined as data collected by one or more of the authors for the purposes of the research. Exclude studies that used clinical records without obtaining patient consent and studies that used data from registries for clinical or quality control purposes, health care administration databases, national census databases, open access databases collected by other researchers, or biobanks. Systematic reviews and meta-analyses are excluded. Articles labelled as editorials, comments, perspectives, research letters, correspondences, case reports and case series, or similar terms, are excluded.

**Yes: study eligible for inclusion in full-text review.Full text inclusion and exclusion criteria screening form**

**Exclude: not human research.** Exclude if the article does not report on human research.

**Exclude: does not use primary data.** Exclude if the article does not report on a study that used primary data collection, defined as data collected by one or more of the authors for the purposes of the research. Exclude studies that used clinical records without obtaining patient consent and studies that used data from registries for clinical or quality control purposes, health care administration databases, national census databases, open access databases collected by other researchers, or biobanks. Systematic reviews and meta-analyses are excluded.

**Exclude: brief report or communication.** Exclude if the article is labelled as an editorial, comment, perspective, research letter, correspondence, case report or case series, brief report, brief communication, or another similar term. For short papers, which are not specified as brief reports in the article, check the issue contents page on the journal website to determine whether it is labelled as a brief communication.

**Include: study eligible for inclusion in the review.**

**Supplement 4: Data Extraction Form**

**Article information**

**First author last name**

[textbox]

**Publication year**

Note: Use ‘Online ahead of print’ for year if not in final format in journal issue with page numbers or article number for online-only journals.

- 2020
- 2021
- 2022
- 2023
- Online ahead of print

**Publication month**

Note: Use 'Online ahead of print' month if not in final format in journal issue with page numbers.

- January
- February
- March
- April
- May
- June
- July
- August
- September
- October
- November
- December

**Journal name**

Note: Please select the journal the article was published in from the dropdown menu.

[drop-down list]

**Funding source(s)**

Note: Please check all that apply; however, if you check “None” or “Not reported” then no others should be checked.

[checkboxes]

- Industry
- Non-industry (e.g., government, not for profit)
- Combined industry and non-industry
- No study funding
- Not reported

**Country of corresponding author**

Note: To find the country of the corresponding author refer to the corresponding authors contact information. If no address is provided, refer to the corresponding author’s first affiliation.

[drop-down list]

**Country where the study participants were recruited**

Note: If participants from more than one country were included, please select 'multiple countries' from the dropdown menu and in the text box list which countries were included in alphabetical order and separated by a semi-colon (e.g., Australia; Canada; United States).

[drop-down list]

**Study design**

Note: Please select the type of study design.

- Intervention study e.g., clinical trials
- Cross-sectional observational study
- Longitudinal cohort study
- Case-control study
- Measurement properties study
- Diagnostic test accuracy study
- Other (please describe): [textbox]

**Study aim**

**Please describe the aim or purpose of the research as stated by the study authors**

Note: Please provide a brief summary of the main aim or objective of the study. For example, "To assess the effectiveness of a self-help psychological intervention in preventing the development of mental disorders among Syrian refugees in Turkey”.

[textbox]

**Population**

**Description of study participants**

Note: Please give a brief description of the specific population of participants included in the study (e.g., "Adolescents with autism.", "Inpatients with psychosis in a psychiatric hospital.", "patients with acute anorexia nervosa and healthy controls.").

**Number of participants included in the study**

Note: This is the total number of participants who were included in the study (not the number engaged in the research, the number eligible, or the number per group). If the number of participants is not reported, please state "NR".

[textbox]

**Setting where participant recruitment and/or data collection took place (select all that apply)**[checkboxes]

- University campus
- Hospital inpatient setting
- Outpatient or community clinics
- Community centres or other community-based organisations
- Internet
- Unclear (please describe): [textbox]
- Other (please describe): [textbox]
- Not reported

**Topic**

**Study topic**

Note: Please briefly describe the topic under investigation. For example, if the topic is ‘Subjective experience and meaning of delusions in psychosis.’, state “Delusions in psychosis” If the topic is "Understanding the substance use of autistic adolescents and adults", state “Substance use of autistic adolescents and adults.” If the topic is "Therapist-supported online remote behavioural intervention for tics in children and adolescents in England (ORBIT)", state “Behavioural intervention for tics in children and adolescents.” If not reported, please state "NR".

[textbox]

**Topic category (select all that apply)**

Note: Please select the category under investigation. Select one category only that reflects the main purpose of the article. If the article fits more than one category, choose the category that is most closely aligned with the primary research question or objective.

[checkboxes]

- Mental health treatments, interventions, or therapies
- Prevalence or burden of mental disorders
- Mental health measurement or screening tool development or validation
- Factors associated with mental disorders or with prognosis of mental disorders
- Trajectory of mental health symptoms or diagnoses over time
- Neuroimaging study e.g., FMRI
- Genetics and biomarkers study
- COVID-19 study
- Other (please describe): [textbox]

**Patient and public engagement**

**Were there any patient authors?**

Note: Select "Yes" if one or more patient authors were included or if there is a group name in the manuscript and patient authors were included as part of the group. Often this information is provided as an author affiliation. If there is a group name, look online to see if individual group members’ names can be accessed and patient authors can be identified. If the group members names cannot be accessed code as "No". For example, if "SPIN team" is listed as an author, look to see if individual team members names can be accessed and if any are described as patient authors. If individual team members’ names cannot be accessed, select "No".

[checkboxes]

- No
- Yes
- Unclear (please describe): [textbox]

[If Yes or Unclear, answer the following question]

**Number of named patient authors or number of grouped patient authors**

Note: Please state the number of patient authors, or the number of patients included as part of an author group.

[textbox]

**Were any patients acknowledged in the acknowledgments section of the manuscript?**

Note: Select "Yes" if patients were acknowledged either individually or as part of a group in the acknowledgements section. Please copy and paste from the acknowledgements section what was reported. Select "No" if there was no reference to patients in the acknowledgements section.

[checkboxes]

- No
- Yes (please copy and paste what was reported): [textbox]
- Unclear (please describe): [textbox]

**Does the article describe engaging patients and/or the public in the research process? (e.g., research planning, conduct, interpretation, dissemination).**

Note: Select “Yes” if patients or the public were engaged in the research process. If patients or the public were engaged as research participants only, please select “No”. Select “Planned” if the researchers indicate they will be engaging patients in future stages of the research (e.g., in the dissemination of the research). Only select “Planned” if the researchers give explicit examples of how patients or the public will be engaged (e.g., patients will work with researchers to develop knowledge translation tools).

Look in the methods section for information on whether patients or the public were engaged in the research or for a specific section dedicated to patient engagement (e.g., “Patient and Public Involvement”). If information on patient engagement is not included in the methods and a specific section on patient engagement is not found, look at the post manuscript material (e.g., acknowledgements).

[checkboxes]

- No – did not report
- Yes
- Planned
- Unclear (please explain): [textbox]
- No – reported no engagement

[If No, stop here. If Yes, Planned, or Unclear, the following questions will be displayed]

**Where in the article was patient or public engagement described?**

Note: If patient or public engagement was described in the main text of the article, select main text only. Select post manuscript material if patient or public engagement was reported in this section only and not in the main text.

[checkboxes]

- Article main text (e.g., methods or specific patient engagement section)
- Post manuscript material (e.g., acknowledgments)

**Patient and public engagement in the research**

**Stage of engagement (select all that apply)**

Note: Please indicate *at what point* in the research process patients or members of the public were engaged. For example, if patients or the public were engaged in research prioritization (e.g., topic selection) or planning, select "research planning". If patients or the public were engaged in research planning and research conduct, select "research planning" and "research conduct”. If unclear, please select "unclear" and explain your reasoning. This option should be used sparingly, and only if you cannot determine at what point in the research process that patients or the public were engaged.

[checkboxes]

- Research planning (e.g., research agenda setting, topic selection or prioritization, designing or reviewing the study protocol, seeking funding)
- Research conduct (e.g., drafting or reviewing study materials, assisting in or planning for recruiting participants and data collection)
- Interpretation of results (e.g., discussing findings, interpreting data, assessing implications, forming recommendations)
- Dissemination of results (e.g., identifying avenues for dissemination, participating in dissemination efforts such as sharing with patient organisations and on social media, presenting study findings to different audiences, developing dissemination tools)
- Unclear (please explain): [textbox]

**Level of engagement**

Note: Please indicate *to what degree*patients or the public were engaged in the research process, e.g., how much influence they had on research decision making.

If unclear, please select "unclear" and explain your reasoning. This option should be used sparingly, and only if you cannot determine to what degree patients or the public were engaged in the research process.

[checkboxes]

- **Consult** (e.g., patient or public opinion was sought on research activities, and the information was used by researchers to make decisions). For example, the distribution of a survey or individual interviews to assess patient or public attitudes, views and preferences towards a research project and associated activities; patient or public focus groups or workshops to discuss research issues and identify potential solutions. This is principally **one-way sharing of information from patients to researchers**; patients are not part of the research team and not involved in transforming their input into decisions.
- **Involve** (e.g., patients or the public work with researchers to advise and provide recommendations on research activities). For example, patient advisory teams, patient committees, or individual patients who provide input into study design and conduct or make recommendations for dissemination strategies. This might involve patient partners on the research team or patients whose advice is sought on a regular basis throughout the study. This would typically involve **two-way communication and input on decisions.** Although patients in this role would not have final responsibility for decision making, they would be involved in decision-making processes and engage in weighing available information and advising on decisions.
- **Partner** (e.g., patients or the public work in partnership with researchers to undertake research and **have decision-making responsibility**). For example, steering committees, boards, or councils where patients or members of the public work with researchers to determine priorities, share and discuss ideas, identify common concerns, and agree on research actions. Patient partners are members of the research team; participate in discussions on how the research will be designed, carried out, or disseminated; and have a role in making decisions.
- Unclear whether Consult or Involve (please explain): [textbox]
- Unclear whether Involve or Partner (please explain): [textbox]

**Comments**

Please record any comments or notes on the article in the box below.

[textbox]

**Supplement 5: Emails to Authors Templates**

**Email Templates**

Email to authors of studies that do not report whether they engaged patients or the public:

Subject: Question on patient engagement in your study in [JOURNAL]

Dear [NAME],

We are conducting a review of patient and public engagement in mental health research. Your study, [TITLE], which was published in [YEAR] in [JOURNAL] is included in the review. However, we could not determine from the article whether you engaged patients or the public in your research process.

**Patient or public engagement involves** working with patients to plan, conduct, interpret, or disseminate research. Examples include distributing surveys or conducting interviews to gain patients or members of the public input into research activities, holding group meetings or discussions for patients to provide their opinion and perspectives on the research, including patients on advisory committees or boards where patients or members of the public work with researchers to share ideas, identify common concerns and agree on research actions.

We would be grateful if you or one of your co-authors could answer a few questions on patient and public engagement in your study via the survey link below. The survey will take no more than 5 minutes to complete.

[INSERT LINK TO QUALTRICS SURVEY]

Thank you, [NAME], in advance, for your help with this.

Email to authors of studies that report engaging patients or the public, but do not report on the stage or level of engagement.

Subject: Question on patient engagement in your study in [JOURNAL]

Dear [NAME],

We are conducting a review of patient and public engagement in mental health research. Your study, [TITLE], which was published in [YEAR] in [JOURNAL] is included in the review. We determined that you engaged patients or the public in your research. However, we were not sure how patients or the public were engaged in the research process.

[INSERT SPECIFIC QUESTIONS REGARDING THE STAGE OR LEVEL OF ENGAGEMENT, AS APPROPRIATE]

Thank you, [NAME], in advance, for your help with this.

**Qualtrics Survey: to authors of studies that do not report whether they engaged patients or the public**

Q1. [textbox]

Thank you, in advance, for your help with our review of patient and public engagement in mental health research.

Your responses will help us to determine current practices for patient and public engagement in high-impact research in psychiatry.

This survey should take no more than 5 minutes to complete.

We **define** **patient or public engagement as** working with patients to plan, conduct, interpret, or disseminate research. Examples include distributing surveys or conducting interviews to gain patients or members of the public input into research activities, holding group meetings or discussions for patients to provide their opinion and perspectives on the research, including patients on advisory committees or boards where patients or members of the public work with researchers to share ideas, identify common concerns and agree on research actions.

Q2. [single response]

**Did your study engage patients or the public in the research process (e.g., in research planning, conduct, interpretation, or dissemination)?** Please select “Yes” if patients or members of the public were engaged in the research process. If patients or members of the public were engaged as research participants only, please select “No”.

☐Yes

☐ No

[If NO, to end of survey]

[If YES, to Q3.]

Q3. [multiple response]

**At what point(s) in the research process were patients or the public engaged?** Please select all that apply.

☐ Research planning (e.g., research agenda setting, topic selection or prioritization, designing or reviewing the study protocol, seeking funding)

☐ Research conduct (e.g., drafting or reviewing study materials, assisting in or planning for recruiting participants and data collection)

☐ Interpretation of results (e.g., discussing findings, interpreting data, assessing implications, forming recommendations)

☐ Dissemination of results (e.g., identifying avenues for dissemination, participating in dissemination efforts such as sharing with patient organisations and on social media, presenting study findings to different audiences, developing dissemination tools)

Q4. [textbox]

**Please explain your response.**

Q5. [single response]

**At what level were patients or the public engaged in the research process?** (e.g., how much influence did they have on decision-making?) Please select the highest level of engagement that applies.

☐ **Consult** (e.g., patient or public opinion was sought on research activities, and the information was used by researchers to make decisions). For example, the distribution of a survey or individual interviews to assess patient or public attitudes, views and preferences towards a research project and associated activities; patient or public focus groups or workshops to discuss research issues and identify potential solutions. This is principally **one-way sharing of information from patients to researchers**; patients are not part of the research team and not involved in transforming their input into decisions.

☐ **Involve** (e.g., patients or the public work with researchers to advise and provide recommendations on research activities). For example, patient advisory teams, patient committees, or individual patients who provide input into study design and conduct or make recommendations for dissemination strategies. This might involve patient partners on the research team or patients whose advice is sought on a regular basis throughout the study. This would typically involve **two-way communication and input on decisions.** Although patients in this role would not have final responsibility for decision making, they would be involved in decision-making processes and engage in weighing available information and advising on decisions.

☐ **Partner** (e.g., patients or the public work in partnership with researchers to undertake research and **have decision-making responsibility**). For example, steering committees, boards, or councils where patients or members of the public work with researchers to determine priorities, share and discuss ideas, identify common concerns, and agree on research actions. Patient partners are members of the research team; participate in discussions on how the research will be designed, carried out, or disseminated; and have a role in making decisions.

Q6. [textbox]

**Please explain your response.**

Q7. [textbox]

End of survey

Thank you for your time taken to complete this survey. Your responses are highly valued.

**Supplement 6: Characteristics of Individual Studies**

An excel file with individual study characteristics and results can be found here:

<https://osf.io/f7rh9>

**Supplement 7: Number of Studies that Engaged People with Lived Experience, Reported in the Article or Based on Author Email Responses, for the Eligible Journals**

| **Journal** | **Included Articles:**  **N** | **Engaged People with Lived Experience:**  **Reported in the Article**  **N** | **Engaged People with Lived Experience:**  **Per Author Report**  **N** | | **Did not Engage People with Lived Experience:**  **Per Author Report**  **N** | **Authors did not Respond**  **N** |
| --- | --- | --- | --- | --- | --- | --- |
| World Psychiatry | 0 | 0 | 0 | 0 | | 0 |
| Lancet Psychiatry | 3 | 1 | 1 | 0 | | 1 |
| JAMA Psychiatry | 4 | 0 | 1 | 0 | | 3 |
| Psychotherapy and Psychosomatics | 1 | 0 | 0 | 1 | | 0 |
| American Journal of Psychiatry | 2 | 0 | 0 | 0 | | 2 |
| Brain Behavior and Immunity | 3 | 0 | 1 | 1 | | 1 |
| Asian Journal of Psychiatry | 13 | 0 | 3 | 6 | | 4 |
| Journal of Anxiety Disorders | 4 | 0 | 0 | 1 | | 3 |
| Journal of Neurology Neurosurgery and Psychiatry | 11 | 0 | 0 | 5 | | 6 |
| Evidence-based Mental Health^a^ | 0 | 0 | 0 | 0 | | 0 |
| Molecular Psychiatry | 15 | 0 | 2 | 5 | | 8 |
| Journal of the American Academy of Child and Adolescent Psychiatry | 1 | 0 | 0 | 0 | | 1 |
| Biological Psychiatry | 6 | 0 | 1 | 4 | | 1 |
| Neuropsychobiology | 5 | 0 | 1 | 2 | | 2 |
| Psychiatry and Clinical Neurosciences | 7 | 0 | 0 | 3 | | 4 |
| Psychiatry Research | 27 | 1 | 2 | 8 | | 16 |
| British Journal of Psychiatry | 2 | 0 | 0 | 2 | | 0 |
| Psychological Medicine | 24 | 0 | 4 | 9 | | 11 |
| International Journal of Social Psychiatry | 13 | 1 | 6 | 5 | | 1 |

^a^Now called BMJ Mental Health.

| **Supplement 8: Number and Percent of Studies that Engaged People with Lived Experience, Reported and Unreported in the Article, by Stages of Engagement and Levels of Engagement** | | | | | | | | | | |
| --- | --- | --- | --- | --- | --- | --- | --- | --- | --- | --- |
|  | **Stage of Engagement** | | | | |  | **Level of Engagement** | | | |
|  | **Planning**  **N (%)** | **Conduct**  **N (%)** | **Interpretation**  **N (%)** | **Dissemination**  **N (%)** | **Did not report**  **N (%)** |  | **Consult**  **N (%)** | **Involve**  **N (%)** | **Partner**  **N (%)** | **Did not report** |
| **All (N = 25)** | 14 (56%) | 13 (52%) | 7 (28%) | 11 (44%) | 5 (20%) |  | 10 (40%) | 7 (28%) | 2 (8%) | 6 (24%) |
| **Region/Country** |  |  |  |  |  |  |  |  |  |  |
| Europe (n = 14) | 8 (57%) | 8 (57%) | 2 (14%) | 5 (36%) | 3 (21%) |  | 5 (36%) | 6 (43%) | 0 (0%) | 3 (21%) |
| North America (n = 2) | 2 (100%) | 1 (50%) | 1 (50%) | 1 (50%) | 0 (0%) |  | 1 (50%) | 0 (0%) | 1 (50%) | 0 (0%) |
| China (n = 5) | 3 (60%) | 2 (40%) | 3 (60%) | 3 (60%) | 1 (20%) |  | 2 (40%) | 1 (20%) | 0 (0%) | 2 (40%) |
| Other Asian countries (n = 3) | 1 (33%) | 2 (67%) | 1 (33%) | 2 (67%) | 0 (0%) |  | 2 (67%) | 0 (0%) | 1 (33%) | 0 (0%) |
| Other countries (n = 1) | 0 (0%) | 0 (0%) | 0 (0%) | 0 (0%) | 1 (100%) |  | 0 (0%) | 0 (0%) | 0 (0%) | 1 (100%) |
| **Study Design** |  |  |  |  |  |  |  |  |  |  |
| Cross-sectional observational study (n = 12) | 7 (58%) | 8 (67%) | 3 (25%) | 4 (33%) | 3 (25%) |  | 4 (33%) | 5 (42%) | 0 (0%) | 3 (25%) |
| Longitudinal cohort study (n = 5) | 2 (40%) | 2 (40%) | 2 (40%) | 3 (60%) | 1 (20%) |  | 3 (60%) | 1 (20%) | 0 (0%) | 1 (20%) |
| Intervention trial (n = 5) | 4 (80%) | 2 (40%) | 1 (20%) | 2 (40%) | 0 (0%) |  | 3 (60%) | 1 (20%) | 1 (20%) | 0 (0%) |
| Case-control study (n = 2) | 1 (50%) | 1 (50%) | 1 (50%) | 1 (50%) | 1 (50%) |  | 0 (0%) | 0 (0%) | 1 (50%) | 1 (50%) |
| Diagnostic assessment study (n = 1) | 0 (0%) | 0 (0%) | 0 (0%) | 1 (100%) | 0 (0%) |  | 0 (0%) | 0 (0%) | 0 (0%) | 1 (100%) |
| Other study design (n = 0) | 0 (0%) | 0 (0%) | 0 (0%) | 0 (0%) | 0 (0%) |  | 0 (0%) | 0 (0%) | 0 (0%) | 0 (0%) |
| **Population** |  |  |  |  |  |  |  |  |  |  |
| Non-psychiatric population (n = 9) | 5 (56%) | 3 (33%) | 3 (33%) | 3 (33%) | 2 (22%) |  | 5 (56%) | 2 (22%) | 0 (0%) | 2 (22%) |
| People with severe mental disorders (n = 8) | 3 (38%) | 5 (62%) | 1 (12%) | 2 (25%) | 3 (38%) |  | 0 (0%) | 4 (50%) | 1 (12%) | 3 (38%) |
| People with common mental disorders (n = 3) | 3 (100%) | 2 (67%) | 2 (67%) | 3 (100%) | 0 (0%) |  | 2 (67%) | 0 (0%) | 1 (33%) | 0 (0%) |
| People with neurological disorders (n = 0) | 0 (0%) | 0 (0%) | 0 (0%) | 0 (0%) | 0 (0%) |  | 0 (0%) | 0 (0%) | 0 (0%) | 0 (0%) |
| People at risk of mental disorders (n = 2) | 1 (50%) | 1 (50%) | 0 (0%) | 1 (50%) | 0 (0%) |  | 2 (100%) | 0 (0%) | 0 (0%) | 0 (0%) |
| People with neurodevelopmental disorders (n = 1) | 0 (0%) | 0 (0%) | 0 (0%) | 1 (100%) | 0 (0%) |  | 0 (0%) | 0 (0%) | 0 (0%) | 1 (100%) |
| Other disorders (n = 2) | 2 (100%) | 2 (100%) | 1 (50%) | 1 (50%) | 0 (0%) |  | 1 (50%) | 1 (50%) | 0 (0%) | 0 (0%) |
| **Study Topic** |  |  |  |  |  |  |  |  |  |  |
| Other factors associated with mental disorders or with prognosis of mental disorders (n = 11) | 6 (55%) | 4 (36%) | 3 (27%) | 4 (36%) | 4 (36%) |  | 4 (36%) | 2 (18%) | 1 (9%) | 4 (36%) |
| Neuroimaging, genetics, or biomarkers study (n = 4) | 3 (75%) | 3 (75%) | 1 (25%) | 2 (50%) | 1 (25%) |  | 2 (50%) | 1 (25%) | 0 (0%) | 1 (25%) |
| Effects of mental health interventions (n = 4) | 3 (75%) | 2 (50%) | 1 (25%) | 1 (25%) | 0 (0%) |  | 2 (50%) | 1 (25%) | 1 (25%) | 0 (0%) |
| Other topic (n = 6) | 2 (33%) | 4 (67%) | 2 (33%) | 3 (50%) | 0 (0%) |  | 2 (33%) | 3 (50%) | 0 (0%) | 1 (17%) |

CI: confidence interval
